# Parental depression and emotional feeding practices are associated with a tendency towards overeating in preadolescents

**DOI:** 10.3389/fnut.2024.1497509

**Published:** 2025-01-03

**Authors:** Catharina Sarkkola, Marja H. Leppänen, Aino-Maija Eloranta, Laura Räisänen, Satu Männistö, Heli Viljakainen

**Affiliations:** ^1^Folkhälsan Research Center, Helsinki, Finland; ^2^Department of Public Health, Faculty of Medicine, University of Helsinki, Helsinki, Finland; ^3^Faculty of Medicine, University of Helsinki, Helsinki, Finland; ^4^Institute of Biomedicine, School of Medicine, University of Eastern Finland, Kuopio, Finland; ^5^Institute of Public Health and Clinical Nutrition, University of Eastern Finland, Kuopio, Finland; ^6^Department of Medicine, Endocrinology and Clinical Nutrition, Kuopio University Hospital, Kuopio, Finland; ^7^Faculty of Medicine and Health Technology (MET), Tampere University, Tampere, Finland; ^8^Department of Pediatrics, Tampere University Hospital, Tampere, Finland; ^9^Finnish Institute for Health and Welfare (THL), Helsinki, Finland

**Keywords:** overeating, eating behavior, children and adolescents, overweight and obesity, parental depression, emotional feeding, parental feeding practices, family

## Abstract

**Background:**

Children’s eating behaviors, including a tendency towards overeating, are strongly influenced by the family. Children prone to overeating are at a high risk of excessive weight gain, which can lead to further adverse health outcomes. Therefore, identifying factors that contribute to overeating is crucial for promoting healthy weight development. Given the inconsistencies in previous research, mostly involving young children, we investigated the child and parental characteristics associated with overeating in preadolescence.

**Methods:**

The cross-sectional study included 5,973 preadolescents aged 9–12 years from the Finnish Health in Teens (Fin-HIT) cohort. A tendency towards overeating was based on a parent-reported question. We utilized extensive parent questionnaire and Medical Birth Register data, and used ordinal and stepwise logistic regression to identify the independent determinants of overeating.

**Results:**

The proportion of preadolescents with a parent-reported tendency towards overeating was 10% (*n* = 606). In the multivariable model, boys had higher odds of overeating (OR 1.30, 95% CI 1.06–1.58) compared with girls. Preadolescents with overweight and obesity had 9- and 30-fold odds (95% CI 7.31–11.29 and 20.07–44.54, respectively) of overeating compared with healthy-weight preadolescents. Furthermore, parental depression and emotional feeding increased the odds of overeating in the preadolescent (OR 1.48, 95% CI 1.08–2.02 and OR 1.27, 95% CI 1.03–1.57).

**Conclusion:**

Along with child weight status and sex, parental depression and emotional feeding were independently associated with overeating in preadolescence. Therefore, it is important to support parents’ mental health and their healthy feeding practices. Our findings can be targeted to manage overeating and prevent overweight in children and adolescents.

## 1 Introduction

Children’s eating behavior is influenced by genetics, environmental factors, and neural mechanisms (1, 2). Particularly the family plays a crucial role in shaping eating behaviors during childhood (3). Overeating, i.e., consuming food beyond nutritional needs increases the risk of excessive weight gain in children and has become a topical concern (4). This behavior may result from low satiety responsiveness (SR), which indicates poor regulation of food intake based on feelings of fullness, high food responsiveness (FR), characterized by eating in response to food cues, or a combination of both (5, 6). Other closely related dimensions include emotional overeating (EOE) and external eating, i.e., eating in response to external cues rather than internal hunger cues. Parental questionnaires are often used to assess these eating behaviors in children.

There is limited knowledge on the frequency of overeating among children. In a Portuguese study involving 6–18-year-olds, 24% were considered overeaters (7). The evidence on factors associated with overeating and its different aspects in children is inconsistent. For example, some studies link early-life factors like breastfeeding to better eating control (high SR, low SR or low EOE) later in childhood (8–10), while others found no such associations (11–15). Being born preterm has been linked to SR but not to other aspects of overeating in preadolescence (8, 16).

Similarly, results on family factors such as parental education, body mass index (BMI), depression, and presence of siblings are contradictory, with studies showing varied outcomes depending on the child’s age and study methodology (8, 11, 16–21). Only one study showed a link between maternal depressive symptoms and child overeating (21). Parental feeding practices may either foster or undermine children’s ability to self-regulate their eating (22). These practices include emotional feeding (using food to calm or cheer up the child) and using food as a reward (23). Some (24, 25) studies demonstrate a link between parental emotional feeding and aspects of overeating, but not all (26). In a clinical study of preadolescents living with overweight, maternal emotional feeding was the key predictor of child emotional eating (27). In addition, several parenting factors, such as low maternal support, but high psychological and behavioral control (28), as well as non-authoritative and emotionally non-responsive parenting (29) have been linked to child emotional eating. Associations regarding food as a reward have also been reported, although the results have not been confirmed for all aspects of overeating in longitudinal study settings (24, 30–33).

Previous studies have identified several early-life and family characteristics that may contribute to overeating in children. However, only a few studies have assessed these factors simultaneously to investigate their hierarchy and independent associations. Most studies have focused on children under school age, leaving a gap in research on preadolescents approaching or experiencing puberty—a unique developmental stage characterized by rapid physical growth, hormonal changes, and emotional shifts that can influence eating behaviors. Our study aimed to determine the prevalence of a parent-reported tendency towards overeating (hereafter, simply ‘overeating’) in Finnish 9–12-year-old preadolescents, and to investigate the associated child and parental characteristics.

## 2 Materials and methods

### 2.1 Study population

The study included participants from the prospective Finnish Health in Teens cohort (Fin-HIT), a school-based study conducted in 2011–2014 (34). This study consists of 11,407 preadolescent children aged 9–12 years and their 6,046 parents, 87% of them mothers, from Finland’s largest cities and their surrounding areas. In this cross-sectional study, we used a subsample of 5,973 preadolescents with available parental-reported data on overeating. The preadolescents’ mean age, sex distribution, prevalence of overweight and obesity, and maternal socioeconomic status (SES) in the subsample were consistent with those of the entire cohort (Supplementary Table 1). Seventy-nine percent of the preadolescents in the subsample participated at their schools, the rest from home. The participants from school and home recruitment had similar characteristics (Supplementary Table 1) and were therefore analyzed together. No one in the cohort has been diagnosed with any rare syndrome (Cushing, Prader-Willi, Bardet-Biedl, Alström) that may influence BMI, according to data from the national Register of Primary Health Care visits (35).

This study was conducted according to the guidelines in the Declaration of Helsinki, and all procedures were approved by the Coordinating Ethics Committee of the Hospital District of Helsinki and Uusimaa (169/13/03/00/10; August 10, 2010). Written informed consent was obtained from the children and one parent per child.

### 2.2 Overeating

One of the parents filled in a questionnaire, mostly online. Overeating was assessed with a question based on The Avon Longitudinal Study of Parents and Children (ALSPAC, UK) questionnaire at 10.5 years (36). The question was: *Do you agree or disagree with this statement: ‘If I did not guide or regulate my child’s eating, s/he would eat too much.’* Answers were scored on a five-point Likert scale: *agree, slightly agree, neither agree nor disagree, slightly disagree, disagree*. The two first and two last response options were combined for the analyses composing three overeating categories (groups): overeating, possible overeating, no overeating.

### 2.3 Child determinants

The parent reported whether the child had two homes or families, siblings living in the main home, or food allergies. Trained fieldworkers measured the preadolescents’ height and weight at school. The preadolescents participating at home provided self-reported measures with an adult’s assistance, which have proven to be accurate for epidemiological studies (37). BMI was calculated (kg/m^2^) and the preadolescents were classified with thinness, healthy weight, overweight or obesity according to the International Obesity Task Force age-and sex-specific cutoffs (38).

Data from birth and early childhood were also included. Information on gestational age at birth was obtained from the Medical Birth Register maintained by the Finnish Institute for Health and Welfare (39). Gestational age was classified as preterm (<37 weeks), full-term (37 + 0–42 + 0 weeks) and post-term (>42 + 0 weeks). Since the development of oral feeding skills has been reported to be delayed among early preterm infants (40), additional analyses for this subgroup were performed using the cutoff <34 weeks (41). In addition, the parent reported whether the child received breastmilk, infant formula or both during the first 6 months of her/his life, and the child’s age when breastfeeding was stopped. Breastfeeding duration was categorized into <1 month, 1–5 months, 6–11 months and ≥12 months based on Finnish breastfeeding recommendations at the time when the children were born (42, 43). Lastly, the parent was also asked about the age at which the child entered daycare.

### 2.4 Parental determinants

Maternal SES at the time of the child’s birth was obtained from the Medical Birth Register and used as a five-class variable (upper-level employees, lower-level employees, manual workers, students and others) as previously reported (44). This register includes information only about the mother giving birth, not the father. The parent (either the mother or father) reported her/his own current height and weight in the questionnaire. BMI was calculated and categorized as thinness (<18.5 kg/m^2^), healthy weight (18.5–24.9 kg/m^2^), overweight (25.0─29.9 kg/m^2^) and obesity (≥30.0 kg/m^2^). The parent also reported whether s/he had been diagnosed or treated for depression or exhaustion/burnout at any time, and whether s/he had experienced an eating disorder, such as anorexia nervosa, bulimia nervosa, or an atypical eating disorder.

The questions on current parental feeding practices included emotional feeding (*‘I cheer her/him up with something to eat if s/he is sad or upset’* with the answer scale *always, sometimes, never*, *not applicable*) and food as a reward (*‘It is OK to offer sweets as a reward for good behavior,’* scored with the five-point Likert scale from *‘agree’* to *‘disagree’*). In the analyses, the categories always and sometimes were combined. For the item ‘food as reward’, the first two and the last two categories were combined to create a three-class variable: yes, no opinion, and no. These two question items were adopted from the ALSPAC Study (36).

### 2.5 Statistical analyses

The child and parental characteristics were compared between the three overeating categories (overeating, possible overeating, no overeating) with the *χ*^2^ test for categorical variables and ANOVA for continuous variables. The unadjusted associations of each determinant with overeating were tested with ordinal logistic regression, resulting in odds ratios (OR) with 95% confidence intervals (CI) for overeating in comparison to the reference categories. All statistically significant (*p* < 0.05) determinants were selected for the stepwise multinomial logistic regression analysis, to define their hierarchy and independent associations with overeating. All these determinants were added in the multivariable model concurrently as stepwise terms with forward entry. The associations were presented with ORs. The third category (no overeating) was used as a reference. There was no interaction by sex between any of the determinants and overeating, so girls and boys were analyzed together. We also tested whether participation at home versus school modified the association between child BMI category and overeating, but we found no proof for interaction. All statistical analyses were performed using the software package IBM SPSS Statistics version 24.0.

### 2.6 Sensitivity analysis

For the sensitivity analysis we considered medications likely having side effects on appetite and/or BMI, i.e., Methylphenidate, Prednisolone, Mirtazapine, Aripiprazole, Risperidone, Chlorpromazine, Olanzapine, Quetiapine, and other antipsychotics, if the medication was ongoing or was estimated to have been finished within 1 month prior to participation. In addition, Thyroxine purchases within 4 months after participation were considered a proxy for latent hypothyroidism. Data on drug purchases were collected from the national Drug Purchase Register, maintained by the Finnish Social Insurance Institution. We also used information on the child’s possible eating disorder, i.e., anorexia nervosa, bulimia nervosa, or an atypical eating disorder, which was obtained from the parents’ questionnaire.

The sensitivity analysis was conducted in a subset of 5,789 participants, after excluding preadolescents with medication (*n* = 154), eating disorder (*n* = 28), or their combinations (*n* = 2). Since the determinants of overeating were the same in the subsample and the whole sample, we used the whole sample of 5,973 preadolescents throughout the analyses.

## 3 Results

### 3.1 Participant characteristics

In the study population of 5,973 preadolescents, 51% were girls (Table 1). The mean age was 11.2 years (standard deviation [SD] 0.8), and 12.1 and 2.4% of the preadolescents were living with overweight and obesity, respectively. The proportion of preadolescents with overeating was 10.1% (*n* = 606), of which 56% were boys. Overweight and obesity were more common within the overeating group than in the possible overeating or no overeating groups. One-third of the preadolescents had a mother with high SES (Table 2), while maternal SES did not differ between the overeating groups. Of the parents, 41% presented as living with overweight or obesity, and this was more common in the overeating group.

**Table 1:** Child characteristics for the total sample and overeating categories are reported as *n* (%), unless noted otherwise, along with their associations with overeating in the unadjusted model.

		Total	Overeating	Possible overeating	No overeating	*χ*^2^ *p*	Overeating			
	Missing	*n* = 5,973	*n* = 606	*n* = 236	*n* = 5,131		OR*	95% CI		*p*
Sex	0					0.001				
Girls		3,072 (51.4)	269 (44.4)	119 (50.4)	2,684 (52.3)		Ref.			
Boys		2,901 (48.6)	337 (55.6)	117 (49.6)	2,447 (47.7)		1.29	1.12	1.50	<0.001
Mean (SD) age, years	34	11.2 (0.8)	11.2 (0.7)	11.1 (0.7)	11.2 (0.8)	0.656†	1.02	0.92	1.12	0.741
Number of homes	13					0.104				
1		5,070 (85.1)	496 (82.4)	196 (83.4)	4,378 (85.5)		Ref.			
2		890 (14.9)	106 (17.6)	39 (16.6)	745 (14.5)		1.23	1.02	1.50	0.035
Living with siblings	0					0.361				
Yes		5,282 (88.4)	531 (87.6)	215 (91.1)	4,536 (88.4)		Ref.			
No		691 (11.6)	75 (12.4)	21 (8.9)	595 (11.6)		0.99	0.79	1.25	0.946
Food allergy	71					0.911				
Yes		943 (16.0)	98 (16.5)	36 (15.4)	809 (15.9)		1.02	0.84	1.25	0.841
No		4,959 (84.0)	496 (83.5)	198 (84.6)	4,265 (84.1)		Ref.			
BMI category‡	172					<0.001				
Thinness		632 (10.9)	9 (1.5)	8 (3.5)	615 (12.3)		0.27	0.16	0.44	<0.001
Healthy weight		4,326 (74.6)	259 (43.9)	147 (63.9)	3,920 (78.7)		Ref.			
Overweight		702 (12.1)	237 (40.2)	65 (28.3)	400 (8.0)		7.45	6.24	8.91	<0.001
Obesity		141 (2.4)	85 (14.4)	10 (4.3)	46 (0.9)		21.88	15.51	30.88	<0.001
Birth and early childhood										
Gestational age	206					0.693				
Preterm§		311 (5.4)	35 (6.1)	15 (6.6)	261 (5.3)		1.19	0.87	1.63	0.263
Full-term		5,295 (91.8)	522 (90.6)	207 (91.2)	4,566 (92.0)		Ref.			
Post-term		161 (2.8)	19 (3.3)	5 (2.2)	137 (2.8)		1.11	0.72	1.72	0.636
Milk type when <6 months	8					0.007				
Breastmilk only		2,627 (44.0)	256 (42.5)	108 (45.8)	2,263 (44.1)		Ref.			
Both		3,163 (53.0)	322 (53.4)	119 (50.4)	2,722 (53.1)		1.01	0.87	1.17	0.880
Infant formula only		138 (2.3)	14 (2.3)	8 (3.4)	116 (2.3)		1.16	0.73	1.86	0.529
Unknown		37 (0.6)	11 (1.8)	1 (0.4)	25 (0.5)		3.22	1.63	6.35	<0.001
Breastfed, months	681					0.336				
< 1		227 (4.3)	24 (4.6)	11 (5.2)	192 (4.2)		1.14	0.77	1.69	0.509
1–5		1,160 (21.9)	127 (24.2)	41 (19.3)	992 (21.8)		1.07	0.86	1.34	0.533
6–11		2,400 (45.4)	221 (42.2)	97 (45.8)	2,082 (45.7)		0.96	0.79	1.16	0.669
≥ 12		1,391 (26.3)	134 (25.6)	57 (26.9)	1,200 (26.3)		Ref.			
Unknown		114 (2.2)	18 (3.4)	6 (2.8)	90 (2.0)		1.69	1.05	2.71	0.030
Entered daycare, years	11					0.091				
Not at all		508 (8.5)	43 (7.1)	13 (5.5)	452 (8.8)		0.80	0.58	1.10	0.171
< 1		705 (11.8)	80 (13.3)	21 (8.9)	604 (11.8)		1.08	0.83	1.41	0.545
1–3		3,493 (58.6)	354 (58.8)	158 (66.9)	2,981 (58.2)		1.10	0.91	1.32	0.338
> 3		1,256 (21.1)	125 (20.8)	44 (18.6)	1,087 (21.2)		Ref.			

*Ordinal logistic regression.

†ANOVA.

‡International Obesity Task Force (38).

§Gestational weeks <37, early preterms considered.

**Table 2:** Parental characteristics for the total sample and overeating categories are reported as *n* (%), unless noted otherwise, along with their associations with overeating in the unadjusted model.

		Total	Overeating	Possible overeating	No overeating	*χ*^2^ *p*	Overeating			
	Missing	*n* = 5,973	*n* = 606	*n* = 236	*n* = 5,131		OR*	95% CI		*p*
Mean (SD) age, years	9	42.4 (5.4)	42.0 (5.4)	42.5 (6.0)	42.5 (5.4)	0.103†	0.99	0.97	1.00	0.072
Socioeconomic status‡	283					0.244				
Upper-level employees		1,885 (33.1)	178 (31.6)	66 (29.2)	1,641 (33.5)		Ref.			
Lower-level employees		2,338 (41.1)	226 (40.1)	91 (40.3)	2,021 (41.2)		1.05	0.88	1.26	0.578
Manual workers		576 (10.1)	66 (11.7)	32 (14.2)	478 (9.8)		1.36	1.06	1.76	0.018
Students		545 (9.6)	57 (10.1)	27 (11.9)	461 (9.4)		1.21	0.93	1.59	0.157
Others§		346 (6.1)	36 (6.4)	10 (4.4)	300 (6.1)		1.04	0.74	1.46	0.821
BMI category	107					<0.001				
Thinness		106 (1.8)	7 (1.2)	3 (1.3)	96 (1.9)		0.79	0.41	1.53	0.486
Healthy weight		3,332 (56.8)	280 (47.0)	107 (46.7)	2,945 (58.4)		Ref.			
Overweight		1,690 (28.8)	192 (32.2)	86 (37.6)	1,412 (28.0)		1.49	1.26	1.76	<0.001
Obesity		738 (12.6)	117 (19.6)	33 (14.4)	588 (11.7)		1.96	1.59	2.41	<0.001
Depression	156					0.012				
Yes		539 (9.3)	74 (12.5)	23 (10.1)	442 (8.8)		1.40	1.11	1.77	0.005
No		5,278 (90.7)	516 (87.5)	204 (89.9)	4,558 (91.2)		Ref.			
Exhaustion or burnout	156					0.839				
Yes		287 (4.9)	28 (4.7)	13 (5.7)	246 (4.9)		1.01	0.72	1.42	0.936
No		5,530 (95.1)	562 (95.3)	214 (94.3)	4,754 (95.1)		Ref.			
Eating disorder	210									
Yes		319 (5.5)	49 (8.3)	13 (5.8)	257 (5.2)	0.007	1.53	1.15	2.04	0.003
No		5,444 (94.5)	539 (91.7)	211 (94.2)	4,694 (94.8)		Ref.			
Feeding practices										
Emotional feeding	10					<0.001				
Always or sometimes		1,998 (33.5)	245 (40.6)	93 (39.4)	1,660 (32.4)		1.41	1.21	1.65	<0.001
Never		3,653 (61.3)	336 (55.6)	123 (52.1)	3,194 (62.3)		Ref.			
Not applicable		312 (5.2)	23 (3.8)	20 (8.5)	269 (5.3)		1.08	0.77	1.51	0.663
Sweets as reward	1					<0.001				
Yes		787 (13.2)	86 (14.2)	39 (16.5)	662 (12.9)		1.22	0.99	1.50	0.061
No opinion		567 (9.5)	58 (9.6)	47 (19.9)	462 (9.0)		1.42	1.13	1.79	0.003
No		4,618 (77.3)	462 (76.2)	150 (63.6)	4,006 (78.1)		Ref.			

*Ordinal logistic regression.

†ANOVA.

‡Maternal socioeconomic status.

§Self-employed, stay-at-home-mothers, unemployed, pensioners.

### 3.2 Determinants of overeating

In the unadjusted model, being a boy, having two homes or families, and living with overweight or obesity were associated with overeating (Table 1). Having a parent with lower SES, overweight or obesity, depression or eating disorder were also linked to overeating (Table 2). Additionally, having a parent either practicing emotional feeding or with a liberal attitude toward sweets as a reward had higher odds of overeating compared with those whose parents were against emotional feeding and sweets as a reward.

**Figure 1:**
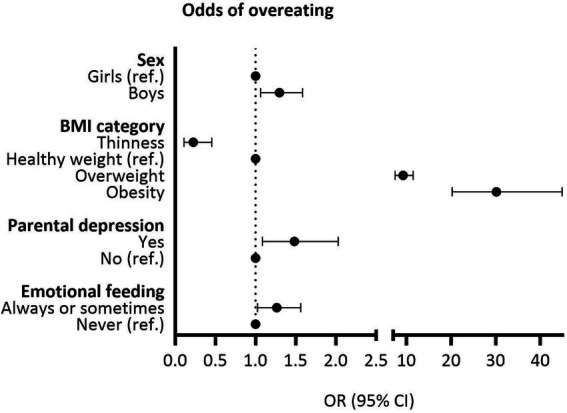
Statistically significant associations (*p* < 0.05) with overeating in the stepwise multivariable model.

In the stepwise multivariable model, boys had higher odds of overeating (OR 1.30, 95% CI 1.06–1.58) than girls (Figure 1). Preadolescents living with overweight and obesity had 9- and 30-fold odds (95% CI 7.31–11.29 and 20.07–44.54, respectively) of overeating, compared with preadolescents with healthy weight. Parental history of depression increased the odds of overeating in the preadolescent (OR 1.48, 95% CI 1.08–2.02). Preadolescents whose parents practiced emotional feeding had higher odds of overeating than preadolescents whose parents did not engage in such practices (OR 1.27, 95% CI 1.03–1.57).

## 4 Discussion

We simultaneously evaluated various early-life and family determinants of a parent-reported tendency towards overeating in a large cohort of Finnish preadolescents. This is the first study to investigate these relationships, and it fulfils the gap of knowledge concerning eating behaviors in preadolescents. There is sparse data on the frequency of overeating among children and adolescents. In our sample, overeating was reported in 10%, which is less than in a Portuguese study with 6─18-year-olds (24%) (7).

Overeating was strongly linked to preadolescent’s overweight and obesity in this study, consistent with previous reports (5, 45). Overeating and its various aspects are considered mediating factors for overweight and obesity (5). For example, low SR is likely to contribute to higher energy intake and it is also one of the mechanisms through which genetic predisposition leads to weight gain (46, 47). In fact, determinants such as parental emotional feeding and rewarding practices have also been connected with overweight (48).

In addition to weight, we identified several independent determinants of overeating. In our study, boys were more prone to overeating than girls. This may be partly explained by differences in parental feeding practices towards sons and daughters (49). Sons are encouraged to eat more and are served larger portions (50, 51), while daughters are taught food avoidant strategies, and parents are in general more concerned about their daughters’ than their sons’ weight status (52, 53).

The finding that parental depression increased the odds of overeating is both interesting and concerning. This might reflect preadolescents’ response to parent ill-health through overeating. Moreover, parents with depression may have altered feeding behaviors and give their children less autonomy in eating (54). Our findings align with results from the ALSPAC study, UK, where maternal depressive symptoms during the first 5 years of the child’s life predicted greater parental worry about child overeating at age 8, and greater self-reported emotional and external eating at age 14 among 3,887 participants (21). The question on overeating in that study was different from ours, as it specifically covered parental worry about child overeating. Moreover, mothers completed a questionnaire on postnatal depression (55), while our question addressed any history of diagnosed and/or treated depression. Contrasting our findings, a Danish study (*n* = 1,939) demonstrated that maternal psychiatric disorders were not associated with child-reported emotional or external eating at ages 11–12 (16). Register data on psychiatric disorders, and The Eating Pattern Inventory for children (EPI-C) (56) were used in the Danish study. No association was found between parental depression and SR or FR either, as measured with the Children’s Eating Behavior Questionnaire (CEBQ) (6) in 2–5-year-old Australian children (*n* = 977) (11). Given that all studies rely on different methodologies, future research should replicate these findings.

Furthermore, we observed that parental emotional feeding was linked to overeating. This finding is plausible, since receiving food for other purposes than hunger might disturb the natural satiety signals, resulting in overeating (57). However, this aspect has been sparsely studied with conflicting results. Previous studies are based on rather small samples with less than 550 children (24–26), and most included much younger children than our study. In line with our findings, an Australian study reported a link between maternal emotional feeding at age 2 and a tendency to overeat at age 3 (24). The researchers used a factor called ‘tendency to overeat’ based on items from the parent version of the Dutch Eating Behavior Questionnaire (58) and CEBQ (6). A US study using CEBQ and including children from low-income and minority households also observed a longitudinal association between emotional feeding and FR, but the results on SR were opposite at different ages (25). Higher emotional feeding at the age of 3 years predicted higher SR 1 year later, but higher emotional feeding at the age of 4 years was associated with lower SR 1 year later. On the other hand, a Chinese study involving 7–12-year-old children did not find associations between emotional feeding and any of these eating behaviors measured by the CEBQ (26).

The main strength of the Fin-HIT study is its large sample size. The sample is representative of school children in urban and semi-urban areas of Finland. Thus, the findings may be generalized to similar populations in areas with moderate population density in Western countries. We included a wide variety of variables from several data sources: parent-reported questionnaire, anthropometric measures made mainly by trained fieldworkers, and objective register data. This allowed us to cover multiple aspects of the topic and address their hierarchy in a systematic way. In addition, a sensitivity analysis was conducted, and it confirmed our findings.

The main limitation relates to the cross-sectional study design: we were unable to indicate causality between the determinants and outcomes. Moreover, we used a single question to assess overeating, instead of a validated questionnaire such as the CEBQ with mean scores for the SR, FR and EOE subscales (6). Our measure did not give a continuous ranking for the children on a scale, but divided them into groups, which might affect the results.

Many determinants were not measured objectively since reported by the parents. We do not have verified diagnoses of parental depression, nor do we know when it manifested. However, our question is considered easy to answer and suitable for epidemiological study purposes. Breastfeeding was reported retrospectively and reflects the situation on average 11 years before estimating overeating. However, it has been proved that breastfeeding can be reliably reported several years later (14). The register data on maternal SES was from the time of the child’s birth. We assume the SES to be similar or higher at the time of data collection; higher in particular among the tenth of the mothers who were previously studying.

Future research should explore the mechanisms underlying the associations between parental depression, emotional feeding, and overeating in preadolescents. Longitudinal studies would allow to establish causal relationships and examine how these factors interact over time. A crucial next step is to investigate how interventions aimed at improving parental mental health, and promoting healthy feeding practices, impact children’s eating behaviors and weight trajectories.

## 5 Conclusion

Our findings highlighted that along with weight status, sex, parental depression, and emotional feeding are linked to a parent-reported tendency towards overeating in preadolescence. Since overeating predisposes to obesity, and childhood obesity is a substantial public health issue, all efforts to recognize the causes of obesity and to prevent it are needed. To promote health, parents should be taught to use other ways than food to console their child. Finally, it is important to support parental mental health since this might affect the child’s eating behavior.

## Data Availability

The data analyzed in this study is subject to the following licenses/restrictions: the datasets are available from the corresponding author on reasonable request. Requests to access these datasets should be directed to Heli Viljakainen, heli.viljakainen@helsinki.fi.

## References

[1] Hughes SO Frazier-Wood AC. Satiety and the self-regulation of food take in children: a potential role for gene-environment interplay. Curr Obes Rep. (2016) 5:81–7. doi: 10.1007/s13679-016-0194-y, PMID: 26847550 PMC4798905

[2] Carnell S Haworth CM Plomin R Wardle J. Genetic influence on appetite in children. Int J Obes. (2008) 32:1468–73. doi: 10.1038/ijo.2008.127, PMID: 18679413

[3] Savage JS Fisher JO Birch LL. Parental influence on eating behavior: conception to adolescence. J Law Med Ethics. (2007) 35:22–34. doi: 10.1111/j.1748-720X.2007.00111.x, PMID: 17341215 PMC2531152

[4] Syrad H Johnson L Wardle J Llewellyn CH. Appetitive traits and food intake patterns in early life. Am J Clin Nutr. (2016) 103:231–5. doi: 10.3945/ajcn.115.117382, PMID: 26675767 PMC4691671

[5] Boutelle KN Manzano MA Eichen DM. Appetitive traits as targets for weight loss: the role of food cue responsiveness and satiety responsiveness. Physiol Behav. (2020) 224:113018. doi: 10.1016/j.physbeh.2020.113018, PMID: 32562711 PMC8020879

[6] Wardle J Guthrie CA Sanderson S Rapoport L. Development of the Children's eating behaviour questionnaire. J Child Psychol Psychiatry. (2001) 42:963–70. doi: 10.1111/1469-7610.00792 11693591

[7] Machado BC Dias P Sousa Lima V Carneiro A Gonçalve S. Frequency and correlates of picky eating and overeating in school-aged children: a Portuguese population-based study. J Child Fam Stud. (2021) 30:1198–213. doi: 10.1007/s10826-021-01936-0

[8] Omar OM Massoud MN Ibrahim AG Khalaf NA. Effect of early feeding practices and eating behaviors on body composition in primary school children. World J Pediatr. (2022) 18:613–23. doi: 10.1007/s12519-022-00559-9, PMID: 35666456 PMC9169027

[9] Reyes M Hoyos V Martinez SM Lozoff B Castillo M Burrows R. Satiety responsiveness and eating behavior among Chilean adolescents and the role of breastfeeding. Int J Obes. (2014) 38:552–7. doi: 10.1038/ijo.2013.191, PMID: 24145926 PMC3981889

[10] Yelverton CA Geraghty AA O'Brien EC Killeen SL Horan MK Donnelly JM. Breastfeeding and maternal eating behaviours are associated with child eating behaviours: findings from the ROLO kids study. Eur J Clin Nutr. (2021) 75:670–9. doi: 10.1038/s41430-020-00764-7, PMID: 32999419 PMC8035071

[11] Boswell N Byrne R Davies PSW. Eating behavior traits associated with demographic variables and implications for obesity outcomes in early childhood. Appetite. (2018) 120:482–90. doi: 10.1016/j.appet.2017.10.012, PMID: 29024677

[12] Hathcock A Krause K Viera AJ Fuemmeler BF Lovelady C Østbye T. Satiety responsiveness and the relationship between breastfeeding and weight status of toddlers of overweight and obese women. Matern Child Health J. (2014) 18:1023–30. doi: 10.1007/s10995-013-1331-9, PMID: 23925718

[13] Higgins RC Keller KL Aruma JC Masterson TD Adise S Fearnbach N. Influence of exclusive breastfeeding on hippocampal structure, satiety responsiveness, and weight status. Matern Child Nutr. (2022) 18:e13333. doi: 10.1111/mcn.13333, PMID: 35167726 PMC9218327

[14] Möller LM de Hoog ML van Eijsden M Gemke RJ Vrijkotte TG. Infant nutrition in relation to eating behaviour and fruit and vegetable intake at age 5 years. Br J Nutr. (2013) 109:564–71. doi: 10.1017/S0007114512001237, PMID: 22717117

[15] Pang WW McCrickerd K Quah PL Fogel A Aris IM Yuan WL. Is breastfeeding associated with later child eating behaviours? Appetite. (2020) 150:104653. doi: 10.1016/j.appet.2020.104653, PMID: 32151607 PMC7347415

[16] Munkholm A Olsen EM Rask CU Clemmensen L Rimvall MK Jeppesen P. Early predictors of eating problems in preadolescence-a prospective birth cohort study. J Adolesc Health. (2016) 58:533–42. doi: 10.1016/j.jadohealth.2016.01.006, PMID: 27107908

[17] Albuquerque G Severo M Oliveira A. Early life characteristics associated with appetite-related eating behaviors in 7-year-old children. J Pediatr. (2017) 180:38–46.e2. doi: 10.1016/j.jpeds.2016.09.011, PMID: 27769552

[18] Ayine P Selvaraju V Venkatapoorna CMK Bao Y Gaillard P Geetha T. Eating behaviors in relation to child weight status and maternal education. Children. (2021) 8:32. doi: 10.3390/children8010032, PMID: 33430408 PMC7826797

[19] Haycraft E Karasouli E Meyer C. Maternal feeding practices and children's eating behaviours: a comparison of mothers with healthy weight versus overweight/obesity. Appetite. (2017) 116:395–400. doi: 10.1016/j.appet.2017.05.033, PMID: 28536055

[20] Kininmonth AR Smith AD Llewellyn CH Fildes A. Socioeconomic status and changes in appetite from toddlerhood to early childhood. Appetite. (2020) 146:104517. doi: 10.1016/j.appet.2019.104517, PMID: 31743696

[21] Doom JR Deer LK Mickel T Infante A Rivera KM. Eating behaviors as pathways from early childhood adversity to adolescent cardiometabolic risk. Health Psychol. (2024) 43:448–61. doi: 10.1037/hea0001340, PMID: 38407101 PMC11263003

[22] McCrickerd K. Cultivating self-regulatory eating behaviours during childhood: the evidence and opportunities. Nutr Bull. (2018) 43:388–99. doi: 10.1111/nbu.12355

[23] Vaughn AE Ward DS Fisher JO Faith MS Hughes SO Kremers SP. Fundamental constructs in food parenting practices: a content map to guide future research. Nutr Rev. (2016) 74:98–117. doi: 10.1093/nutrit/nuv061, PMID: 26724487 PMC4892304

[24] Rodgers RF Paxton SJ Massey R Campbell KJ Wertheim EH Skouteris H. Maternal feeding practices predict weight gain and obesogenic eating behaviors in young children: a prospective study. Int J Behav Nutr Phys Act. (2013) 10:24. doi: 10.1186/1479-5868-10-24, PMID: 23414332 PMC3582584

[25] Berge JM Miller J Veblen-Mortenson S Kunin-Batson A Sherwood NE French SA. A bidirectional analysis of feeding practices and eating behaviors in parent/child dyads from low-income and minority households. J Pediatr. (2020) 221:93–8.e20. doi: 10.1016/j.jpeds.2020.02.001, PMID: 32247517 PMC7252585

[26] Qiu C Hatton R Li Q Xv J Li J Tian J. Associations of parental feeding practices with children's eating behaviors and food preferences: a Chinese cross-sectional study. BMC Pediatr. (2023) 23:84. doi: 10.1186/s12887-023-03848-y, PMID: 36800939 PMC9938626

[27] Braden A Rhee K Peterson CB Rydell SA Zucker N Boutelle K. Associations between child emotional eating and general parenting style, feeding practices, and parent psychopathology. Appetite. (2014) 80:35–40. doi: 10.1016/j.appet.2014.04.017, PMID: 24780349

[28] Snoek HM Engels RC Janssens JM van Strien T. Parental behaviour and adolescents' emotional eating. Appetite. (2007) 49:223–30. doi: 10.1016/j.appet.2007.02.004 17391806

[29] Topham GL Hubbs-Tait L Rutledge JM Page MC Kennedy TS Shriver LH. Parenting styles, parental response to child emotion, and family emotional responsiveness are related to child emotional eating. Appetite. (2011) 56:261–4. doi: 10.1016/j.appet.2011.01.007, PMID: 21232566

[30] Jansen E Williams KE Mallan KM Nicholson JM Daniels LA. Bidirectional associations between mothers' feeding practices and child eating behaviours. Int J Behav Nutr Phys Act. (2018) 15:3. doi: 10.1186/s12966-018-0644-x, PMID: 29325557 PMC5765660

[31] Steinsbekk S Belsky J Wichstrøm L. Parental feeding and child eating: an investigation of reciprocal effects. Child Dev. (2016) 87:1538–49. doi: 10.1111/cdev.12546, PMID: 27154834

[32] Jansen PW Derks IPM Mou Y van Rijen EHM Gaillard R Micali N. Associations of parents' use of food as reward with children's eating behaviour and BMI in a population-based cohort. Pediatr Obes. (2020) 15:e12662. doi: 10.1111/ijpo.12662, PMID: 32548949 PMC7583369

[33] Roberts L Marx JM Musher-Eizenman DR. Using food as a reward: an examination of parental reward practices. Appetite. (2018) 120:318–26. doi: 10.1016/j.appet.2017.09.024, PMID: 28951237

[34] Figueiredo RAO Simola-Ström S Rounge TB Viljakainen H Eriksson JG Roos E. Cohort profile: The Finnish health in teens (Fin-HIT) study: a population-based study. Int J Epidemiol. (2019) 48:23–4. doi: 10.1093/ije/dyy189, PMID: 30212855 PMC6380305

[35] THL. (2024). Register of primary health care visits. Available at: https://thl.fi/en/web/thlfi-en/statistics-and-data/data-and-services/register-descriptions/register-of-primary-health-care-visits (Accessed December 17, 2024).

[36] Boyd A Golding J Macleod J Lawlor DA Fraser A Henderson J. Cohort profile: the ‘children of the 90s’—the index offspring of the Avon Longitudinal Study of Parents and Children. Int J Epidemiol. (2013) 42:111–27. doi: 10.1093/ije/dys064, PMID: 22507743 PMC3600618

[37] Sarkkola C Rounge TB Simola-Ström S von Kraemer S Roos E Weiderpass E. Validity of home-measured height, weight and waist circumference among adolescents. Eur J Pub Health. (2016) 26:975–7. doi: 10.1093/eurpub/ckw133, PMID: 27578829

[38] Cole TJ Lobstein T. Extended international (IOTF) body mass index cut-offs for thinness, overweight and obesity. Pediatr Obes. (2012) 7:284–94. doi: 10.1111/j.2047-6310.2012.00064.x, PMID: 22715120

[39] THL. (2024). Medical birth register. Available at: https://thl.fi/en/web/thlfi-en/statistics-and-data/data-and-services/register-descriptions/newborns (Accessed December 17, 2024).

[40] Lau C. Development of infant oral feeding skills: what do we know? Am J Clin Nutr. (2016) 103:616s–21s. doi: 10.3945/ajcn.115.109603, PMID: 26791183 PMC4733254

[41] Engle WA Tomashek KM Wallman C. "Late-preterm" infants: a population at risk. Pediatrics. (2007) 120:1390–401. doi: 10.1542/peds.2007-2952, PMID: 18055691

[42] Sairanen S. Imetys Suomessa 1995. Lääkärilehti. (1997) 52:3057.

[43] Hasunen K Kalavainen M Keinonen H Lagström H Lyytikäinen A Nurttila A. Lapsi, perhe ja ruoka. Imeväis- ja leikki-ikäisten lasten, odottavien ja imettävien äitien ravitsemussuositus. Sosiaali- ja terveysministeriön julkaisuja. (2004) 11.

[44] Lommi S Engberg E Tuorila H Kolho KL Viljakainen H. Sex-and weight-specific changes in the frequency of sweet treat consumption during early adolescence: a longitudinal study. Br J Nutr. (2021) 126:1592–600. doi: 10.1017/S0007114521001112, PMID: 33787473 PMC8524426

[45] Kininmonth A Smith A Carnell S Steinsbekk S Fildes A Llewellyn C. The association between childhood adiposity and appetite assessed using the child eating behavior questionnaire and baby eating behavior questionnaire: a systematic review and meta-analysis. Obes Rev. (2021) 22:e13169. doi: 10.1111/obr.13169, PMID: 33554425

[46] Emond JA Tovar A Li Z Lansigan RK Gilbert-Diamond D. FTO genotype and weight status among preadolescents: assessing the mediating effects of obesogenic appetitive traits. Appetite. (2017) 117:321–9. doi: 10.1016/j.appet.2017.07.009, PMID: 28712975 PMC5569318

[47] Llewellyn CH Trzaskowski M van Jaarsveld CHM Plomin R Wardle J. Satiety mechanisms in genetic risk of obesity. JAMA Pediatr. (2014) 168:338–44. doi: 10.1001/jamapediatrics.2013.4944, PMID: 24535189 PMC3981891

[48] Beckers D Karssen LT Vink JM Burk WJ Larsen JK. Food parenting practices and children's weight outcomes: a systematic review of prospective studies. Appetite. (2021) 158:105010. doi: 10.1016/j.appet.2020.105010, PMID: 33075443

[49] Keller KL Kling SMR Fuchs B Pearce AL Reigh NA Masterson T. A biopsychosocial model of sex differences in Children's eating behaviors. Nutrients. (2019) 11:682. doi: 10.3390/nu11030682, PMID: 30909426 PMC6470823

[50] Orrell-Valente JK Hill LG Brechwald WA Dodge KA Pettit GS Bates JE. "Just three more bites": an observational analysis of parents' socialization of children's eating at mealtime. Appetite. (2007) 48:37–45. doi: 10.1016/j.appet.2006.06.006, PMID: 17000028 PMC2045650

[51] Bouhlal S McBride CM Ward DS Persky S. Drivers of overweight mothers' food choice behaviors depend on child gender. Appetite. (2015) 84:154–60. doi: 10.1016/j.appet.2014.09.024, PMID: 25300916 PMC4976487

[52] Tiggemann M Lowes J. Predictors of maternal control over children's eating behaviour. Appetite. (2002) 39:1–7. doi: 10.1006/appe.2002.0487 12160560

[53] Phares V Steinberg AR Thompson JK. Gender differences in peer and parental influences: body image disturbance, self-worth, and psychological functioning in preadolescent children. J Youth Adolesc. (2004) 33:421–9. doi: 10.1023/B:JOYO.0000037634.18749.20

[54] Goulding AN Rosenblum KL Miller AL Peterson KE Chen YP Kaciroti N. Associations between maternal depressive symptoms and child feeding practices in a cross-sectional study of low-income mothers and their young children. Int J Behav Nutr Phys Act. (2014) 11:75. doi: 10.1186/1479-5868-11-75, PMID: 24935753 PMC4072610

[55] Cox JL Holden JM Sagovsky R. Detection of postnatal depression. Development of the 10-item Edinburgh postnatal depression scale. Br J Psychiatry. (1987) 150:782–6. doi: 10.1192/bjp.150.6.782 3651732

[56] Schacht M Richter-Appelt H Schulte-Markwort M Hebebrand J Schimmelmann BG. Eating pattern inventory for children: a new self-rating questionnaire for preadolescents. J Clin Psychol. (2006) 62:1259–73. doi: 10.1002/jclp.20300, PMID: 16897691

[57] Birch LL. Learning to eat: behavioral and psychological aspects. Nestle Nutr Inst Workshop Ser. (2016) 85:125–34. doi: 10.1159/000439503, PMID: 27088340

[58] Braet C Van Strien T. Assessment of emotional, externally induced and restrained eating behaviour in nine to twelve-year-old obese and non-obese children. Behav Res Ther. (1997) 35:863–73. doi: 10.1016/S0005-7967(97)00045-4, PMID: 9299807

