# Supplementary material for: Parental depression and emotional feeding practices are associated with a tendency towards overeating in preadolescents
Source: Front Nutr. 2025 Jan 3;11:1497509. doi: 10.3389/fnut.2024.1497509 (PMC11738931; doi:10.3389/fnut.2024.1497509)
Supplement: Supplementary file 1 [file Table_1.docx]

| **Suppl. Table 1.** Comparisons of participant characteristics between the whole Fin-HIT cohort, subsample of home participants and subsample with parental-reported data. | | | | | | | | | | | | | | | | | | | | | | | | | | | | | | | | | | |
| --- | --- | --- | --- | --- | --- | --- | --- | --- | --- | --- | --- | --- | --- | --- | --- | --- | --- | --- | --- | --- | --- | --- | --- | --- | --- | --- | --- | --- | --- | --- | --- | --- | --- | --- |
|  | | | Whole cohort | | | | | | | | | | | |  | Subsample with parental-reported data | | | | | | | | | | | | | | |  |  |  |  |
|  | | All | | | | | |  | Home participants | | | | | | |  | All | | | | | |  | | | Home participants | | | | | |  |  |  |
|  | | n=11 407 | | |  | | |  | n=1599 | | |  | | | |  | n=5973 | | |  | | |  | | | n=1265 | | | | | |  |  |  |
|  | |  | | |  | | |  | (14.0% of whole cohort) | | | | | | |  |  | | |  | | |  | | | (21.2% of subsample) | | | | | | | |  |
|  | n | | | % | |  | n | | | % | | |  | | n | | | % | | |  | | | n | | | % | | | |  |  |  |  |
| **Sex** |  | | |  | |  |  | | | |  | | |  | |  | | |  | | |  | | |  | | | |  | | | |  |  |
| Girls | 5981 | | | 52.4 | |  | 825 | | | 51.6 | | |  | | 3072 | | | 51.4 | | |  | | | 646 | | | | 51.1 | |  |  |  |  |  |
| Boys | 5423 | | | 47.6 | |  | 774 | | | 48.4 | | |  | | 2901 | | | 48.6 | | |  | | | 619 | | | | 48.9 | |  |  |  |  |  |
| *Missing* | 3 | | |  | |  | 0 | | |  | | |  | | 0 | | |  | | |  | | | 0 | | | |  | |  |  |  |  |  |

**Mean (SD) age, years** 11.2 (0.8) 11.2 (0.1) 11.2 (0.8) 11.2 (0.1)

| *Missing* | 95 |  |  | 0 | |  |  | | | 34 |  | |  | | 0 | | | | |  | | |  |  |  |  |
| --- | --- | --- | --- | --- | --- | --- | --- | --- | --- | --- | --- | --- | --- | --- | --- | --- | --- | --- | --- | --- | --- | --- | --- | --- | --- | --- |
| **BMI category**^a^ |  |  |  |  | |  |  | | |  |  | |  | |  | | | | |  | | |  |  |  |  |
| Thinness | 1175 | 11.0 |  | 112 | | 8.7 |  | | | 632 | 10.9 | | |  | | 99 | | | 8.3 | | |  |  |  |  |  |
| Healthy weight | 7854 | 73.8 |  | 963 | | 74.5 |  | | | 4326 | 74.6 | | |  | | 895 | | | 74.7 | | |  |  |  |  |  |
| Overweight | 1343 | 12.6 |  | 183 | | 14.2 |  | | | 702 | 12.1 | | |  | | 172 | | | 14.4 | | |  |  |  |  |  |
| Obesity | 274 | 2.6 |  | 34 | | 2.6 |  | | | 141 | 2.4 | | |  | | 32 | | | 2.7 | | |  |  |  |  |  |
| *Missing* | 761 |  |  | 307 | |  |  | | | 172 |  | | |  | | 67 | | |  | | |  |  |  |  |  |
| **Parental sex** |  |  |  |  | |  |  | | |  |  | | |  | |  | | |  | | |  |  |  |  |  |
| Female | 8568 | 86.8 |  | 1563 | | 98.1 |  | | | 5299 | 88.9 | | |  | | 1240 | | | 98.0 | | |  |  |  |  |  |
| Male | 1308 | 13.2 |  | 31 | | 1.9 |  | | | 660 | 11.1 | | |  | | 25 | | | 2.0 | | |  |  |  |  |  |
| *Missing* | 59^b^ |  |  | 5 | |  |  | | | 14 |  | | |  | | 0 | | |  | | |  |  |  |  |  |
| **Maternal SES** |  |  |  |  | |  |  | | |  |  | | |  | |  | | |  | | |  |  |  |  |  |
| Upper-level employees | 3169 | 29.7 |  | 421 | | 28.3 |  | | | 1885 | 33.1 | | |  | | 337 | | | 28.6 | | |  |  |  |  |  |
| Lower-level employees | 4299 | 40.3 |  | 663 | | 44.5 |  | | | 2338 | 41.1 | | |  | | 536 | | | 45.4 | | |  |  |  |  |  |
| Manual workers | 1279 | 12.0 |  | 185 | | 12.4 |  | | | 576 | 10.1 | | |  | | 139 | | | 11.8 | | |  |  |  |  |  |
| Students | 1127 | 10.6 |  | 134 | | 9.0 |  | | | 545 | 9.6 | | |  | | 102 | | | 8.6 | | |  |  |  |  |  |
| Others^c^ | 803 | 7.5 |  | 87 | | 5.8 |  | | | 346 | 6.1 | | |  | | 66 | | | 5.6 | | | |  |  |  |  |
| *Missing* | 730 |  |  | 109 | |  |  | | | 283 |  | | |  | | 85 | | |  | | | |  |  |  |  |
| ^a^International Obesity Task Force (Cole & Lobstein, 2012)  ^b^Base for calculation: 9935 parents with consent | | | | |  | | |  |  | |  |  | | | | |  |  | | |  | | | |  | |
| ^c^Self-employed, stay-at-home-mothers, unemployed, pensioners | | | | | | | | | | |  |  | | | | |  |  | | |  | | |  | |  |
